# RNA Não Codificante e Hipertrofia Cardíaca

**DOI:** 10.36660/abc.20240698

**Published:** 2024-11-13

**Authors:** Gustavo Augusto Ferreira Mota, Mariana Gatto, Claudia Maria Silva Cyrino, Silvio Assis de Oliveira, Marina Politi Okoshi

**Affiliations:** 1 Universidade Estadual Paulista Faculdade de Medicina de Botucatu Departamento de Clínica Médica São Paulo SP Brasil Departamento de Clínica Médica, Faculdade de Medicina de Botucatu, Universidade Estadual Paulista (UNESP), São Paulo, SP – Brasil; 2 Universidade Estadual Paulista Faculdade de Medicina de Botucatu Departamento de Enfermagem São Paulo SP Brasil Departamento de Enfermagem, Faculdade de Medicina de Botucatu, Universidade Estadual Paulista (UNESP), São Paulo, SP – Brasil; 3 Universidade Federal de Mato Grosso do Sul Instituto Integrado de Saúde Campo Grande MS Brasil Instituto Integrado de Saúde, Universidade Federal de Mato Grosso do Sul (UFMS), Campo Grande, MS – Brasil

**Keywords:** Sistema Renina-Angiotensina, Remodelação Ventricular, Doenças Cardiovasculares, Biologia de Sistemas

Apesar de avanços na medicina cardiovascular, as doenças cardíacas constituem grave problema de saúde pública devido a sua grande prevalência, custos elevados e alta morbimortalidade.^1^ Portanto, há necessidade de melhorar a compreensão sobre mecanismos moleculares envolvidos na fisiopatologia das doenças cardiovasculares.^2^

Após um estímulo agressor, o coração passa pelo processo de remodelação, inicialmente caracterizado por mudanças na expressão do genoma. As alterações levam a modificações moleculares, celulares e intersticiais que se manifestam clinicamente por variações no tamanho, forma e função do coração. A remodelação cardíaca é habitualmente acompanhada por ativação de sistemas neuro-hormonais. Em condições fisiológicas, o sistema renina-angiotensina desempenha papel fundamental na regulação da pressão arterial e equilíbrio hidro-eletrolítico. Entretanto, quando excessivamente ativado, induz efeitos deletérios ao coração, entre os quais se destacam a hipertrofia e morte miocitária e fibrose miocárdica intersticial.^3^

Os ácidos ribonucleicos (RNAs), responsáveis pela síntese proteica, são classificados em codificantes (mRNA) e não codificantes (ncRNAs). Os ncRNAs modulam os RNAs codificantes e podem ser subdivididos em longos (lncRNA) e curtos (miRNAs). Os lncRNAs têm sido arbitrariamente definidos como aqueles que apresentam mais que 200 nucleotídeos. Somente uma minoria dos lncRNAs tiveram seus mecanismos de atuação descritos na literatura.^4,5^ O lncRNA CCAT2 foi primeiramente identificado como um oncogene no câncer colorretal; posteriormente, seu papel em outros tipos de câncer como o de pulmão foi relatado.^6^ Poucos autores avaliaram sua função em doenças cardiovasculares.^7^ A sinalização Wnt/β-catenina é necessária para o desenvolvimento e sobrevida embrionária; sua excessiva ativação tem sido associada a fibrose miocárdica. Recentemente, foi sugerida a existência de uma via de sinalização celular envolvendo CCAT2/Wnt/β-catenina com papel na fisiopatologia da injúria miocitária pós-isquêmica.^8^

Na edição atual dos Arquivos Brasileiros de Cardiologia, Zhang et al.^9^ analisaram o envolvimento do lncRNA CCAT2 em dois modelos experimentais: células H9c2, que simulam modelo de cardiomiócitos isolados, tratadas com angiotensina II (AngII), e camundongos submetidos a estenose aórtica transversa. Neste estudo interessante, os autores verificaram que supressão da ação do CCAT2 atenuou alterações in vitro induzidas por Ang II e hipertrofia miocárdica induzida por sobrecarga pressórica. A redução do CCAT2 diminuiu a sinalização Wnt/β-catenina, e a suplementação de LiCl, agonista da sinalização Wnt/β-catenina, restaurou o efeito pró-hipertrófico. Adicionalmente, o inibidor do CCAT2 atenuou fibrose intersticial e a expressão de BNP, ANP e cadeia pesada de β-miosina, e melhorou o desempenho ventricular.

Hipertrofia cardíaca e deposição de colágeno intersticial são alterações que, frequentemente, precedem a disfunção ventricular. Os resultados do estudo sugerem que a via de sinalização CCAT2/Wnt/β-catenina tem papel importante no desenvolvimento de remodelação cardíaca patológica e que silenciamento de CCAT2 tem efeito protetor na injúria induzida por ativação do sistema renina-angiotensina ou sobrecarga pressórica. Terapêuticas experimentais com ncRNA podem ter como alvo outros ncRNAs ou RNAs mensageiro convencionais, apresentar seletividade superior à de medicamentos convencionais, e abordar vias de sinalização celular ainda inexploradas.^10^ Portanto, estudos futuros podem auxiliar no esclarecimento do papel de lncRNAs no processo de remodelação cardíaca patológica.
