# Supplementary material for: RNA Não Codificante e Hipertrofia Cardíaca
Source: Arq Bras Cardiol. 2024 Nov 13;121(10):e20240698. [Article in Portuguese] doi: 10.36660/abc.20240698 (PMC11634219; doi:10.36660/abc.20240698)
Supplement: Translation

# Translation: Long Non-Coding RNA CCAT2 and Pathological Cardiac Remodeling

**DOI:** 10.36660/abc.20240698i

**Authors:** Gustavo Augusto Ferreira Mota, Mariana Gatto, Claudia Maria Silva Cyrino, Silvio Assis de Oliveira, Marina Politi Okoshi

**Affiliations:** 1 São Paulo SP Brazil Departamento de Clínica Médica, Faculdade de Medicina de Botucatu, Universidade Estadual Paulista (UNESP), São Paulo, SP – Brazil; 2 São Paulo SP Brazil Departamento de Enfermagem, Faculdade de Medicina de Botucatu, Universidade Estadual Paulista (UNESP), São Paulo, SP – Brazil; 3 Campo Grande MS Brazil Instituto Integrado de Saúde, Universidade Federal de Mato Grosso do Sul (UFMS), Campo Grande, MS – Brazil

**Keywords:** Renin-Angiotensin System, Ventricular Remodeling, Cardiovascular Diseases, Systems Biology

Despite advances in cardiovascular medicine, heart disease is a serious public health problem due to its high prevalence, elevated cost, and high morbidity and mortality.^1^ Therefore, it is necessary to improve the understanding of molecular mechanisms involved in the pathophysiology of cardiovascular disease.^2^

Following cardiac injury, the heart undergoes a remodeling process, initially characterized by alterations in the genome expression. The changes lead to molecular, cellular, and interstitial modifications that manifest clinically as variations in the size, shape, and function of the heart. Cardiac remodeling is usually accompanied by activation of neurohormonal systems. Under physiological conditions, the renin-angiotensin system plays a fundamental role in regulating blood pressure and fluid-electrolyte balance. However, when excessively activated, it induces deleterious effects on the heart, such as myocyte hypertrophy and death and interstitial myocardial fibrosis.^3^

Ribonucleic acids (RNAs), responsible for protein synthesis, are classified as coding (mRNA) and non-coding (ncRNAs). ncRNAs modulate coding RNAs; they are subdivided into long (lncRNA) and short (miRNAs). The lncRNAs have been arbitrarily defined as those with more than 200 nucleotides. Only a minority of lncRNAs has had their action mechanisms described in the literature.^4,5^ The lncRNA CCAT2 was first identified as an oncogene in colorectal cancer; later, its role in other cancers, such as lung cancer, was reported.^6^ Few authors have evaluated the role of the lncRNA CCAT2 in cardiovascular diseases.^7^ Wnt/β-catenin signaling is required for embryonic development and survival; its excessive activation has been associated with myocardial fibrosis. Recently, a cellular signaling pathway involving CCAT2/Wnt/β-catenin has been suggested to play a role in the pathophysiology of post-ischemic myocyte injury.^8^

In the current edition of the *Arquivos Brasileiros de Cardiologia*, Zhang et al.^9^ analyzed the involvement of the lncRNA CCAT2 in two experimental models: H9c2 cells, which simulate isolated cardiomyocytes, treated with angiotensin II (AngII), and mice subjected to transverse aortic stenosis. In this interesting study, the authors observed that suppression of CCAT2 action attenuated in vitro changes induced by Ang II and myocardial hypertrophy caused by pressure overload. CCAT2 suppression decreased Wnt/β-catenin signaling, and LiCl supplementation, an agonist of Wnt/β-catenin signaling, restored the pro-hypertrophic effect. Additionally, the CCAT2 inhibitor attenuated interstitial fibrosis and the expression of BNP, ANP, and β-myosin heavy chain and improved ventricular performance.

Cardiac hypertrophy and interstitial collagen deposition often precede ventricular dysfunction. The results of the study suggest that the CCAT2/Wnt/β-catenin signaling pathway plays an important role in the development of pathological cardiac remodeling and that CCAT2 silencing has a protective effect on injury induced by activation of the renin-angiotensin system or pressure overload. Experimental ncRNA therapeutics may target other ncRNAs or conventional messenger RNAs, exhibit superior selectivity to conventional drugs, and address unexplored cellular signaling pathways.^10^ Therefore, future studies are needed to clarify the role of lncRNAs in pathological cardiac remodeling.
